# Probabilistic dietary exposure assessment of polycyclic aromatic hydrocarbons (PAHs) and its associated disease burden in Singapore

**DOI:** 10.1038/s41598-026-39906-5

**Published:** 2026-02-12

**Authors:** Angela Li, Min Ern Chen, Geraldine Songlen Lim, Benjamin Er, Mei Hui Liu, Valerie Sin, Wei Jie Seow, Gene Yong-Kwang Ong, R Ponampalam, Boon Kiat Kenneth Tan, Joanna Khoo, Joanne Sheot Harn Chan, Kyaw Thu Aung

**Affiliations:** 1 National Centre for Food Science, Singapore Food Agency, 7 International Business Park, Singapore, 609919 Singapore; 2 Faculty of Science Department of Food Science & Technology, National University of Singapore, 2 Science Drive, Singapore, 117543 Singapore; 3 Saw Swee Hock School of Public Health, National University of Singapore and National University Health System, 12 Science Drive 2, Singapore, 117549 Singapore; 4 Department of Medicine, Yong Loo Lin School of Medicine, National University of Singapore and National University Health System, 10 Medical Drive, Singapore, 117597 Singapore; 5 Department of Emergency Medicine, KK Women’s and Children’s Hospital, SingHealth-Duke NUS Paediatrics Academic Clinical Programme, Duke-NUS Medical School, 8 College Road, Singapore, 169857 Singapore; 6 Department of Emergency Medicine, Singapore General Hospital, Outram Road, Singapore, 169608 Singapore; 7 School of Biological Sciences, Nanyang Technological University, 60 Nanyang Drive, Singapore, 637551 Singapore

**Keywords:** Polycyclic aromatic hydrocarbons, Food safety, food contaminants, probabilistic dietary exposure, Cancer risk, Disease burden, Diseases, Environmental sciences, Health care, Risk factors

## Abstract

Polycyclic aromatic hydrocarbons (PAHs) are process contaminants with dietary exposure as a key route into the human body. Findings from the Singapore Total Diet Study revealed that higher levels of PAHs were found in food categories such as nuts and seeds, sauces and condiments, fruiting vegetables as well as fungi and seaweed. The choice of cooking method may influence the PAH levels in food such as fish and seafood products where stir-frying was associated with higher PAHs levels than boiling and steaming. Monte Carlo simulation was applied in the PAHs probabilistic exposure assessment using optimistic and pessimistic model scenarios. Lifetime cancer risk due to total dietary exposure to PAHs was estimated to be between 4.63 × 10^− 5^ and 5.17 × 10^− 3^. Attributable Disability-Adjusted Life Years (DALYs) for the Singapore population were estimated to be between 2.36 × 10^− 1^ and 92.5 years under the optimistic and pessimistic model scenarios respectively. These values reflect a relatively low burden of disease attributable to dietary PAHs exposure for consumers in Singapore and elsewhere. Further research and monitoring, along with policy considerations, will be crucial for reducing dietary exposure to PAHs and safeguarding consumer health.

**Supplementary Information:**

The online version contains supplementary material available at 10.1038/s41598-026-39906-5.

## Introduction

Polycyclic aromatic hydrocarbons (PAHs) belong to a large group of organic compounds consisting of two or more fused aromatic rings, which are generated by incomplete combustion or pyrolysis of organic matter, as well as from industrial processes^1^. Present in air, soil, water as well as the food chain, PAHs bioaccumulate and exhibit prolonged ecological presence due to their strong lipophilicity and environmental persistence^2,3^. Given their carcinogenic, mutagenic, and genotoxic effects, exposure to PAHs can pose a major public-health concern^4,5^.

There are various pathways for human exposure to PAHs such as ingestion, inhalation or dermal absorption where the consumption of food is a key route of exposure particularly for non-smokers^1,4,6,7^. The formation of PAHs can also occur during industrial processing and domestic cooking practices involving high-temperature methods such as grilling, roasting, and smoking^1,8,9^.

High levels of PAHs have been reported in filter feeders such as bivalves (mussels and oysters) as well as grilled and barbecued foods^10^. Other studies have identified higher PAH levels in food categories such as meat and fish products, oils and fats, cereals and dry foods^1,9,11,12^. In Singapore, total diet study (TDS) findings revealed PAHs detection across several food categories including fish and seafood, fats and oils, some fruits and vegetables as well as sauces and condiments^13^.

The sustained interest in PAHs over the years reflects their pervasive contamination of environments and toxicity risks to humans. Among PAHs, benzo[a]pyrene (BaP) is of particular concern and is classified by the International Agency for Research on Cancer (IARC) as carcinogenic to humans (Group 1). Several other PAHs are classified by IARC as probably (Group 2 A) or possibly carcinogenic to humans (Group 2B)^14^. The sum of BaP, chrysene (Ch), benzo[b]fluoranthene (BbF), and benz[a]anthracene (BaA) (denoted as PAH4) and/or the sum of PAH4, benzo[k]fluoranthene, benzo[ghi]perylene, dibenz[a, h]anthracene, and indeno[1,2,3-cd]pyrene (denoted as PAH8) are commonly used to indicate levels of PAH contamination. Although PAH8 captures a broader PAHs exposure, it offers minimal additional benefit over PAH4, and maximum levels under European Union regulations for PAHs in food (Regulation (EU) 2023/915) remain based on PAH4 rather than PAH8^12^.

Intake of foods with elevated PAH levels has been associated with higher risks of breast cancer and lung/tracheal cancer^15,16^. Although the occurrence levels of PAHs in commonly consumed foods has been determined from the Singapore’s TDS, the population dietary exposure to PAHs was not evaluated, and specifically the associated disease burden. We aimed to address this gap by characterising dietary exposure to PAHs in Singapore by (i) quantifying PAHs in commonly consumed food, (ii) assessing the influence of cooking methods on PAH levels in food and (iii) evaluating associated cancer risk and burden of disease. Findings from this study will refine exposure prioritisation, support food safety risk assessment, and inform risk mitigation strategies.

## Methods

### Sample selection and food consumption data

Singapore’s Total Diet Study (TDS) was carried out in 2021–2023 as described by Lim et al.^13^. Briefly, the TDS consisted of a review of chemical hazards in food, food consumption survey based on 24-hour recall interviews, food sampling and preparation with in-house domestic cooking methods and analytical testing of food samples. The selection of foods in the TDS included commonly consumed foods by the Singapore population. Samples were purchased from supermarkets, wet markets, e-commerce platforms, and specialty shops such as bakeries and fruit stalls. This paper focused on 21 food categories spanning 264 foods, with a total of 480 composite samples analysed. Food categories analysed in the TDS included but were not limited to grain and grain-based products, meat and meat products, fish and seafood, vegetables, fruits, milk and dairy products. Foods were prepared using multiple cooking methods where relevant (e.g., steaming, frying, boiling), resulting in a larger sample number than the number of foods. Selection of these cooking methods leveraged on survey results and expert consultation to ensure representativeness of Singaporean cooking practices. Each composite sample comprised 15 sub-samples that varied in brand, variety, country of origin, or purchase location to represent typical consumer choices. The sub-samples were then prepared to a consumption-ready state and pooled into a single composite sample, providing a closer estimate of chemical exposure from food consumption. Further details on the TDS sampling methodology are described by Lim et al.^13^. PAHs were among one of the chemical hazard groups included in the TDS where the samples were analysed for BaP, Ch, BbF, and BaA. A 24-hour recall method was used to obtain food consumption data representative of the population in Singapore, where a total of 2,000 participants stratified across different age and ethnic groups were recruited for the survey^13,17^. The TDS was approved by the National Centre for Food Science’s Project Review Committee (Project ID RAP21.1) of the Singapore Food Agency. Verbal informed consent was obtained from all participants in the study. Individual responses were anonymised through the use of an identifier to maintain the anonymity of all participants. All methods were performed in accordance with the relevant guidelines and regulations.

### Analytical testing of samples for PAHs

Analytical testing of the TDS samples was performed in-house at the National Centre of Food Science, which is Singapore’s national food safety testing laboratory accredited under the Singapore Accreditation Council - Singapore Laboratory Accreditation Scheme (SAC-SINGLAS). PAH standards and 13 C-labelled standards for BaP, Ch, BbF and BaA were sourced from AccuStandard Inc. (New Haven, CT, USA), Cerilliant (a MilliporeSigma company, Round Rock, TX, USA) and Chiron AS (Trondheim, Norway). Briefly, the samples were spiked with internal standard mix solution and extracted using HPLC-grade acetonitrile and isopropanol purchased from Tedia (Fairfield, OH, USA). This was followed by sample clean up using HyperSep™ Retain PEP cartridges from Thermo Fisher Scientific (Ward Hill, MA, USA). The eluent was subsequently concentrated under nitrogen, reconstituted in acetonitrile and analysed using gas chromatography tandem mass spectrometry (GC-MS/MS). Samples with high fat content were further cleaned up using hexane before injection. GC-MS/MS analysis was performed using the TSQ 8000 Evo Triple Quadrupole Gas Chromotaphy-Mass Spectrometer/Mass Spectrometer from Thermo Fisher Scientific (Ward Hill, MA, USA). Separation was done using the DB-EUPAH column (30 m × 0.25 mm ID × 0.25 μm) from Agilent Technologies (Santa Clara, CA, USA). The analytical method was fully validated prior to use, with the method validation parameters and limits of detection (LODs) and quantification (LOQs) described by Lim et al.^13^.

### Probabilistic estimation of dietary exposure

The Monte Carlo simulation methodology was adapted from the European Food Safety Authority (EFSA) guidance on probabilistic modelling^18,19^. For each round of the simulation, a person-day (represented by a single day of food consumption for one individual) and the corresponding diet consisting of food items and consumption amounts (g/kg body weight (bw) were randomly selected.

**Fig. 1:**
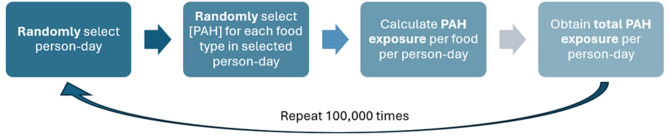
Monte Carlo simulation process.

PAH concentrations were determined from the laboratory analysis of food items identified from the food consumption survey and expressed as PAH4. Due to the inherent varying distribution of PAH concentrations in food, the PAH concentration (mg/kg) for each food item was randomly selected to reflect this natural variability. The PAH exposure (mg/kg bw/day (d)) from that food item was calculated from the multiplication of the consumption amount with the PAH concentration. Summation of the exposures was then performed to derive the total PAH exposure from a 24-hour diet in a person-day. This entire Monte Carlo simulation process was repeated 100,000 times to capture a large enough range of plausible combinations of food consumption amounts and PAH concentrations of the population, and to achieve a stable frequency distribution. The entire process was carried out on Jupyter Notebook with Python Version 3.11.9, as summarised in Fig. 1.

Two model scenarios (optimistic and pessimistic) were simulated using the Monte Carlo method, with each employing a distinct approach to estimating PAH concentrations^20^. These scenarios were simulated to account for the plausible range of PAH concentrations in food. For both scenarios, food consumption amounts were referenced from the food consumption survey and those food items not consumed by the respondents were recorded as 0 g/kg bw.

(i) Optimistic model scenario run.

In this scenario, a key uncertainty arose from data points where PAH concentrations were not detected from laboratory analysis. To account for this, assumptions were made that are expected to generate underestimates of exposure^18^. As such, data points with PAH concentrations not detected from the laboratory analysis were assigned with the lower bound of the limit of detection, that is 0 µg/kg.

(ii) Pessimistic model scenario run.

Conversely, in this scenario, the key uncertainty of undetected PAH concentrations was addressed using assumptions that are expected to generate overestimates of exposure^18^. Therefore, data points with PAH concentrations not detected from the laboratory analysis were assigned with the upper bound of the limit of detection, that is 0.15 µg/kg.

The total PAH concentration used for downstream analysis for each food item was calculated as PAH4 as per Eq. (1). The individual contributions of each analyte to the total PAH concentration was not investigated in this study. Therefore, the specific disease condition(s) associated with each PAH (denoted as $\:{PAH}_{i}$) and its attributable risk were not determined in this study.

1 $$\:\left[PAH4\right]=\:{\sum\:}_{i}^{4}\left[PAHi\right]$$

The probabilistic Monte Carlo simulation model for both optimistic and pessimistic scenario runs relied on the assumptions of independence and stationarity^21^. Independence assumes that the data points are uncorrelated, where dietary intake patterns and consumption amounts are independent across individuals (i.e., the food consumed in any day by any person is not dependent on the foods consumed by another person). This is ensured during the data collection process of the food consumption survey, where participants were randomly selected to ensure that food choices would not be influenced by convenience (e.g. members of the same family are excluded in the survey). This allows variability to stem purely from inherent randomness^22^. Stationarity assumes that PAH concentrations and dietary consumption patterns remain constant over time, unaffected by seasonal or regional variations^23^.

### Cancer risk estimation

Carcinogenic risk from PAHs exposure was calculated according to Eq. (2)^24^. Details on the input values for Eq. (2) are shown in Table 1. The exposure levels of PAHs were obtained as the median of the frequency distributions generated from the two model scenarios (Table 2).

2 $$\:Cancer\:risk\:from\:PAH=\:\frac{{exposure}_{PAH}\:*{SF}_{avg}\:*\:{exposureTime}_{>18y/o}\:}{{expectancy}_{SG}}$$

**Table 1:** Components of Eq. (2).

Symbol	Term	Value		Reference
$\:{Exposure}_{PAH}$	PAH4 exposure	Refer to Table 2		Frequency distribution of Monte Carlo Simulation
$\:{SF}_{avg}$	Slope factor	Benz[a]anthracene	1.00 × 10^− 1^	^25^
		Benzo[a]pyrene	1.00 × 10^1^	
		Benzo[b]fluoranthene	1.00 × 10^− 1^	
		Chrysene	1.00 × 10^− 3^	
$\:{Expectancy}_{SG}$	Life expectancy of resident population in Singapore	83 years		^26^
$\:{exposureTime}_{>18y/o}$	Exposure time	65 years *		-

^*^Obtained from the difference between life expectancy of resident population in Singapore and minimum respondent age of 18 years old.

### Estimated burden of disease

The burden of disease attributable to PAHs (Attributable DALY_PAHCancer_) was estimated using Eq. (3), based on the total DALYs for all cancers in the Singapore population in 2021^27^. The Population Attributable Fraction (PAF) for all cancers due to PAH exposure (PAF_PAH cancer_), is a measure of association that represents the proportion of observed cases of cancer that can be linked to dietary exposure of PAH^28^. PAF_PAH cancer_ was derived according to Eq. (4).

3 $$\:{Attributable\:DALY}_{PAHCancer}={PAF}_{PAH\:cancer}*\:({YLD}_{all\:cancers}+{YLL}_{all\:cancers})$$

Prevalence of dietary exposure to PAHs in Eq. (4) was estimated by applying K-means clustering to the frequency distribution of data points generated from the Monte Carlo simulation^29,30^. Two clusters were obtained as proxies for the exposed and unexposed populations. The difference in exposure levels between the clusters was assessed by the Mann-Whitney U test for non-parametric analysis. The assumptions of the Mann-Whitney U test – non-normality and unequal variance – were verified using the Shapiro-Wilk test and Levene’s test, respectively^31^.

4 $$\:{PAF}_{PAH\:cancer}=\frac{{P}_{PAH\:exposure}\:*\:({RR}_{PAH\:cancer}-1)}{{P}_{PAH\:exposure}*\:\left({RR}_{PAH\:cancer}-\:1\right)+1}$$

The risk ratio of developing cancer over an individual’s lifetime from PAHs exposure (RR_PAH cancer_) was derived according to Eq. (5). The lifetime cancer risk for all cancers in Eq. (5), whereby an individual is at risk of developing cancer at any point during his or her lifetime, was taken to be 0.25, estimated based on empirical data on cancer rates in the Singapore population^32^.

5 $$\:{RR}_{PAH\:cancer}=\frac{{R}_{exposed\:to\:PAH}}{{Total\:cancer\:risk-\:R}_{exposed\:to\:PAH}}$$

Since PAH concentrations in each food item was calculated as PAH4, the disease burden in this study was estimated using DALYs for all cancers. This approach was taken because PAH4 represents a combined measure of four carcinogenic and genotoxic PAHs, as evaluated by JECFA, rather than individual PAHs with specific health effects^1,20^. Given the lack of a precise breakdown of each PAH’s concentration, it was not possible to attribute the burden to specific cancers. Instead, all cancers were used as the reference disease category, reflecting the well-established carcinogenic nature of PAHs.

## Results

### PAHs occurrence in food

**Fig. 2:**
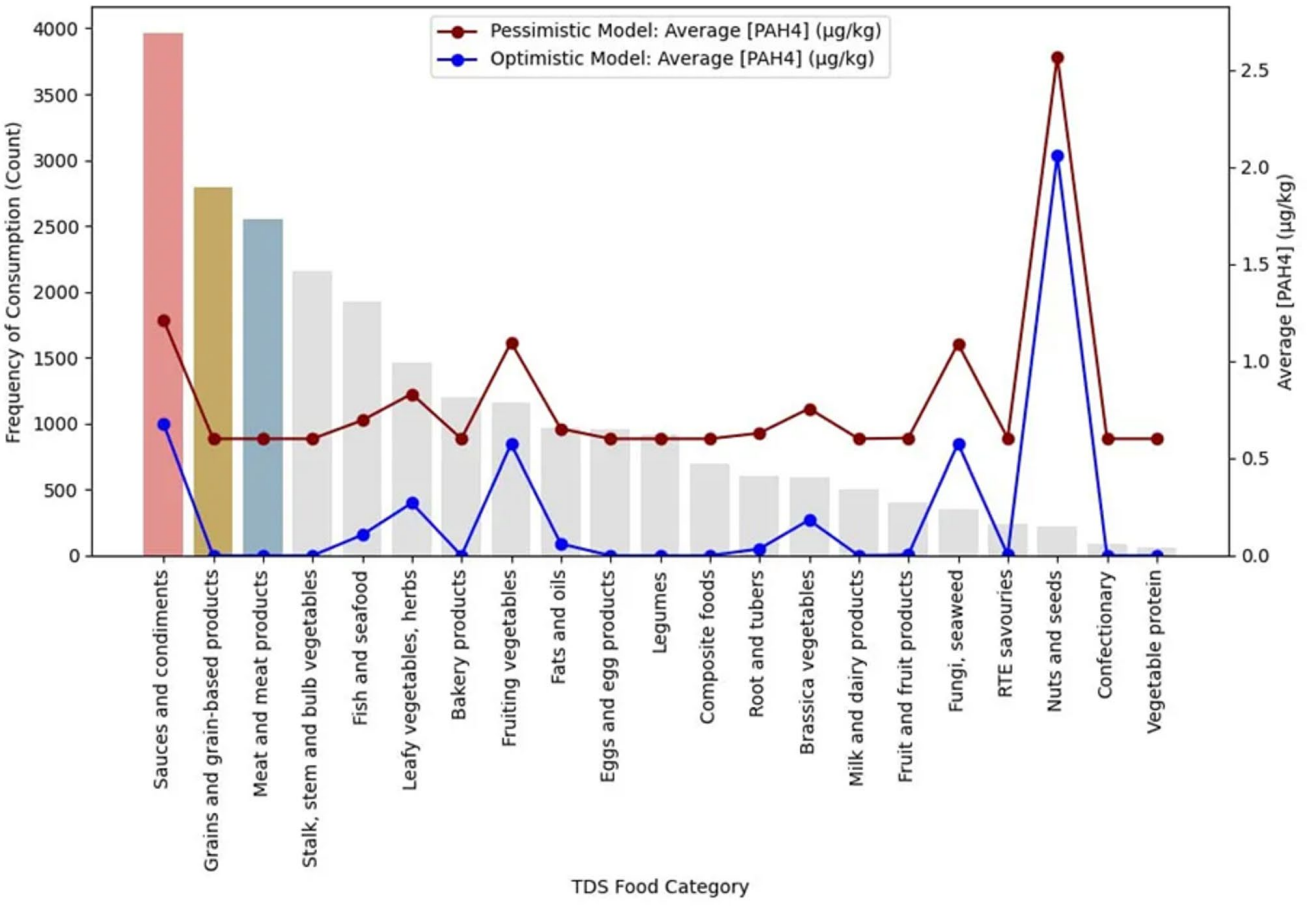
Average PAH4 concentration ([PAH4]) by consumption frequency and food category.

A total of 21 food categories, representing the commonly consumed foods in the Singapore population, were analysed for the presence of 4 PAHs: BaP, Ch, BbF, and BaA, and expressed as PAH4. Foods that require cooking were subjected to multiple cooking methods used in the TDS before the samples were pooled for laboratory analysis. The dietary patterns of the Singapore population assessed in this study are based on those previously described by Lim et al.^13^. With reference to Fig. 2, the top three most consumed food categories from the food consumption survey were sauces and condiments, grains and grain-based products as well as meat and meat products. The highest average PAH4 concentrations derived for each food category were 2.06 µg/kg and 2.57 µg/kg for the optimistic and pessimistic model scenarios respectively. A similar trend was observed for the average PAH4 using the optimistic and pessimistic scenarios with the difference in the magnitude of the values. Higher levels of PAHs were found in the nuts and seeds, the sauces and condiments, the fruiting vegetables as well as the fungi and seaweed categories, specifically in peanut butter products, pepper, chilli and mushroom respectively. Fruiting vegetables refer to warm season annual or perennial plants grown for fruits, and some also for leaves with commodities of tomato, peppers and eggplant as some of the most widely grown fruiting vegetables^33^.

### Influence of cooking method on PAHs in food

**Fig. 3:**
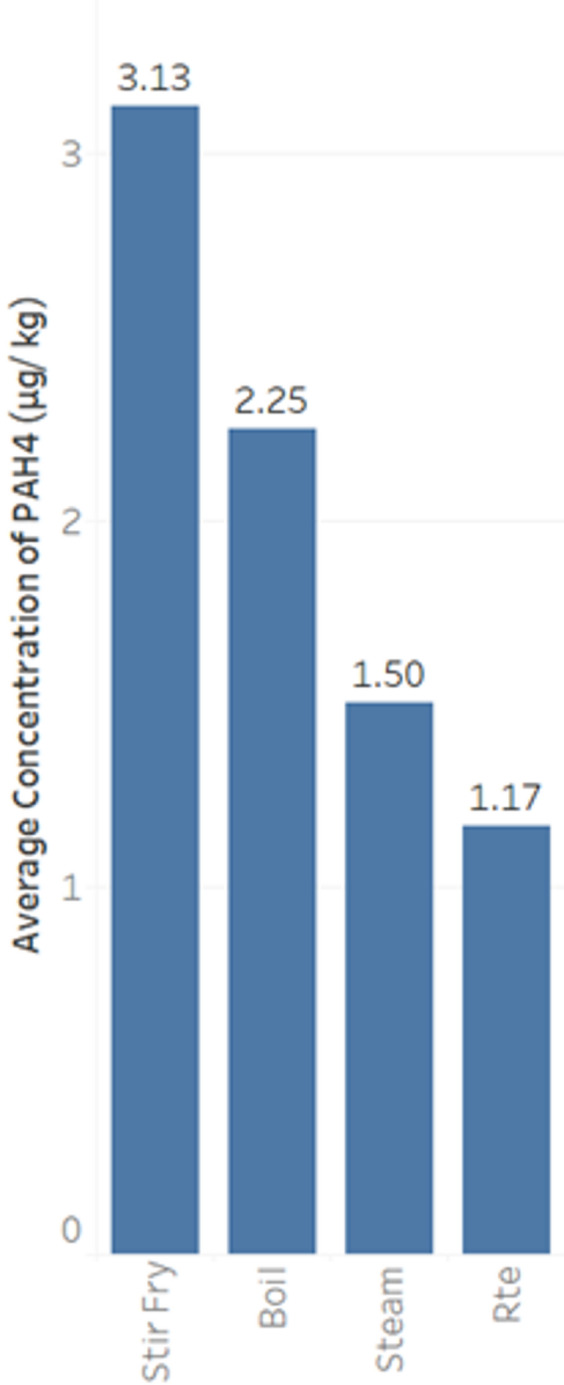
Average PAH4 concentration by cooking method for fish and seafood (Rte denotes ready to eat).

The influence of cooking methods on PAH formation in food was examined in food categories commonly prepared by multiple cooking methods, specifically the animal-sourced protein categories of meat and meat products as well as fish and seafood, from the Singapore TDS data. PAHs were not detected in the meat and meat products category. By contrast, for fish and seafood products, stir-frying was found to have higher levels of average PAH4 than other methods of cooking such as boiling and steaming (Fig. 3). A Kruskal-Wallis test was conducted to assess differences in PAH4 concentrations (µg/kg) across cooking methods. The analysis revealed statistically significant differences between cooking methods (*p* < 0.05), indicating that the cooking method significantly influenced PAH4 concentrations.

### Monte Carlo simulation

**Fig. 4:**
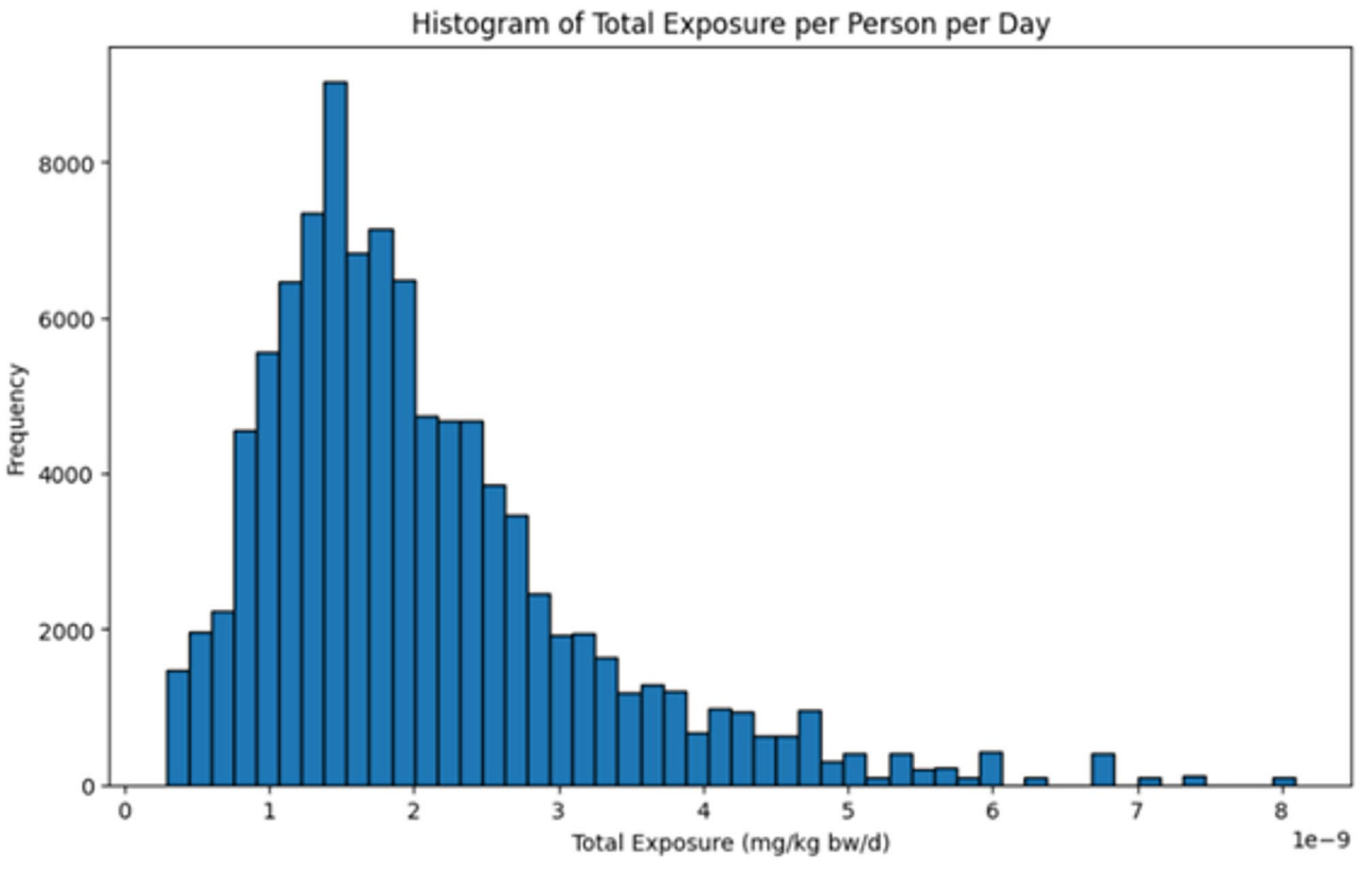
Histogram of Monte Carlo distribution for pessimistic model scenario.

The Monte Carlo simulation was run multiple times under both model scenarios for consistency. Both models yielded comparable ranges of PAHs exposure between the simulation runs, although their distribution characteristics also differed between scenarios. The optimistic model scenario had high kurtosis (34.2), suggestive of a leptokurtic distribution with extreme outliers (Table 2). Conversely, the pessimistic model scenario had lower kurtosis (2.49), which indicated a closer to normal distribution of exposure across the population (Table 2; Fig. 4)^34^.

**Table 2:** Statistical distribution of sample frequency from Monte Carlo simulation of optimistic and Pessimistic model scenarios.

Scenario	PAHs Exposure Range (mg/kg bw/d)	Kurtosis	Proportion of Positive Exposure (%)
Optimistic Model	Min: 0 Max: 1.62 × 10^− 2^	$\:34.2$	8.85
Pessimistic Model	Min: 5.09 × 10^− 3^ Max: 8.05 × 10^− 2^	$\:2.49$	30.6

The largest difference between the models was found between the proportion of population considered with positive PAHs exposure (8.85% and 30.6% for optimistic and pessimistic model scenarios, respectively). As described in the method, K-means clustering was used to categorise individuals into exposed and unexposed groups, and the Mann-Whitney U test confirmed a statistically significant difference in exposure levels between these clusters (*p* < 0.05).

### Cancer risk associated with PAHs dietary exposure

A proposed Minimal Risk Level (MRL) of 4.84 × 10^− 4^ mg/kg bw/day of PAH4 was derived by applying the MRL of BaP from the IARC, along with linear scaling of the intermediate Toxic Equivalency Factors (TEF) for the other three PAH analytes to obtain their respective MRLs. This threshold value falls between the derived PAH exposure level of 1.97 × 10^− 4^ and 2.20 × 10^− 2^ mg/kg bw/day for the optimistic and pessimistic model scenarios respectively (Table 3).

**Table 3:** Estimates of PAH exposure, lifetime cancer risk and total population dalys based on 50th percentile of Monte Carlo frequency distributions.

Scenario	PAHs Exposure (mg/kg bw/d)	Lifetime Cancer Risk due to PAH (probability)	Total Population DALY (years)
Optimistic Model	1.97 × 10^− 4^	4.63 × 10^− 5^	2.36 × 10^− 1^
Pessimistic Model	2.20 × 10^− 2^	5.17 × 10^− 3^	92.5

Consequently, the lifetime absolute cancer risk due to total dietary exposure to PAHs was estimated to be between 4.63 × 10^− 5^ and 5.17 × 10^− 3^, where between 4 in 100,000 and 5 in 1,000 people with the same dietary exposure to PAHs would develop cancer over a lifetime.

### Estimated burden of disease

The PAF for all cancers attributed to PAHs was estimated to be between 1.64 × 10^− 5^ (optimistic model scenario) and 6.44 × 10^− 3^ (pessimistic model scenario). Consequently, attributable DALYs for the Singapore population were estimated to be between 2.36 × 10^− 1^ and 92.5 years under the optimistic and pessimistic model scenarios respectively.

## Discussion

Leveraging a comprehensive TDS covering the commonly consumed food groups in the population, we observed sauces and condiments, grains and grain-based products as well as meat and meat products as the most commonly consumed food categories. Higher intake of sauces and condiments in the typical Singapore diet aligns with the 2022 National Nutrition Survey which reported increased salt consumption, consistent with salt’s frequent use in savoury sauces and condiments^35^. Cereal grains as well as meat and meat products are generally major food categories widely consumed globally and locally, hence they can serve as major dietary routes of human PAHs exposure^2,36,37^. Nonetheless, this TDS design is limited by its reliance on 24-hour recalls that capture short-term intake rather than habitual dietary patterns. Future TDS will consider a two-pronged approach by combining 24-hour recalls with food frequency questionnaires to provide a more comprehensive characterisation of food consumption in Singapore^13^.

In this study, PAH4 levels in food, including the more conservative pessimistic model scenario, were generally low or comparable to similar food categories reported in publications and other TDS studies which reported PAH4 levels up to 57 µg/kg^20,38^. Although the Codex Alimentarius Commission has not established PAH limits for food, several authorities such as Brazil, Canada, China, European Union and Hong Kong have set maximum levels of BaP and/or PAHs on selected foods^9,20^. Under the EU regulation 2023/915, the maximum levels of PAH4 in food, excluding food meant for infants and young children, ranged from 10 to 50 µg/kg, and are well above the average PAH4 levels reported in this study.

Elevated PAH levels were also observed in selected food categories such as nuts and seeds, fruiting vegetables as well as fungi and seaweed. Environmental pollution and exposure to high-temperature roasting likely contribute to heightened levels of PAHs detected in peanuts and peanut oil^39–41^. Similarly, environmental pollution or the heating and drying processes used for spice products could result in higher levels of PAHs content in spices, including pepper^20,42–45^. Additionally, the occurrence of PAHs in red peppers has been found to be related to the drying method used during processing^46^ which could be one of the reasons for the higher levels of PAHs found in dried chilli and mushroom products. Besides, food can be contaminated by environmental PAHs through air (via deposition), soil (via transfer) and water (via deposition and transfer), facilitated by the waxy surface of fruits and vegetables which can concentrate low molecular mass PAHs through surface adsorption, while high molecular mass PAHs bound to particles may contaminate the surface due to atmospheric deposition^1,10^.

It is well documented that PAHs can be formed during food processing, such as smoking or drying, as well as during food preparations under elevated temperatures and/or open flames, such as grilling, toasting, roasting, and frying^9,47^. These cooking methods, which are often influenced by behaviours such as cultural preferences and individual cooking habits, can significantly affect PAH formation in food^47^. In this study, the influence of cooking methods on PAH formation in food was not evident across both the food categories examined, consistent with prior findings indicating insufficient evidence of PAH formation arising from common domestic cooking techniques^48^. While the specific mechanism behind the formation of PAHs in food is lacking, a few possible mechanisms have been proposed: (i) high temperatures (such as grilling, roasting, pan-frying) promote pyrolysis of organic matter such as lipids and proteins in food, that can promote PAHs formation; (ii) fat dripping onto flame generates volatile PAHs which in turn deposits on food; (iii) incomplete combustion of fuel further modulate PAH adherence onto food surfaces^36,49^. On the contrary, moist-heat methods such as boiling, steaming, utilise comparatively lower temperatures with minimal or no smoke generation and food surface charring, thereby resulting in lower PAHs formation in food. In general, studies have shown that PAH levels are reduced in food when they are not subjected to elevated cooking temperature and cooking over direct flame^2^.

The use of probabilistic estimation approach to simulate dietary PAH exposure was done in consideration of the variability in individual diets and PAH concentrations in food^50^. Monte Carlo simulations were selected as the primary method due to their effectiveness in handling uncertainty and variability in complex exposure models^51^. This approach allows for direct random sampling from input distributions to generate multiple possible outcomes, making it particularly suitable for scenarios with significant parameter uncertainty, such as food consumption patterns and PAH concentrations. The method also provides a comprehensive range of potential exposure estimates, crucial for identifying high-risk individuals or subgroups within the population^51^. While EFSA notes that probabilistic methods complement rather than replace deterministic approaches in dietary exposure estimation^19^, Monte Carlo simulation remains valuable for its ability to closely approximate real-world PAH exposure by simulating various food combinations and their corresponding PAH concentrations^18,24^.

The optimistic and pessimistic model scenarios provide counterfactual contexts to simulate the prevalence of dietary PAH exposure. This aligns with counterfactual analysis methods used in some chemical risk assessments applied in previous studies, which evaluate the impact of a factor by comparing real-world conditions to hypothetical alternative scenarios where that factor is altered or removed^52^. By modelling these contrasting exposure conditions, the potential range of PAH exposure in the population and its implications for risk assessment can be assessed.

Uncertainty in dietary consumption and concentrations of PAHs associated with it were mitigated through implementing a large number of iterations (i.e. 100,000 times) in the Monte Carlo simulation^53^, thus resulting in a stable Gaussian Distribution. By the Law of Large Numbers^54^, the large number of iterations produces a sample statistic that converges to the true population statistic of acute dietary exposure. Independence between the random samples was ensured through the TDS study design, where participants were selected at random from the general Singapore population, controlling for dependency in the diet between individuals^13^.

The use of two model scenarios, optimistic and pessimistic, was implemented to encompass the plausible range of PAHs exposure levels through dietary consumption^18^. If the exposure estimate from the optimistic model scenario exceeds the MRL (i.e. the daily PAH exposure unlikely to cause adverse health effects), the actual exposure is likely to be even higher, making risk reduction measures necessary^55,56^. As the optimistic model scenario provides the lowest possible estimate, true exposure can only be equal to or greater than this value. Conversely, if the exposure estimate from the pessimistic model scenario falls below the MRL, actual exposure is likely to be even lower. Since the pessimistic model scenario assumes higher plausible undetected PAH concentrations, its estimates represent an upper bound. If even such an estimate is below the MRL, it suggests there is little concern for excessive PAH exposure. However, continual monitoring of PAH exposure is recommended to detect deviations in exposure levels from the baseline over time.

Various studies on the assessment of PAH exposures in urban populations reported the median Incremental Lifetime Cancer Risk (ILCR) attributable to PAH dietary intake ranged from 6.65 × 10^− 5^ to 1.57 × 10^− 3^, encompassing the range of values estimated in this study^24,57,58^. While the dietary PAH exposure levels derived from the pessimistic model scenario lies above the MRL and may raise concerns, its derived cancer risk remains low.

The PAF for all cancers attributed to PAHs and attributable DALYs observed for the Singapore population reflect an extremely low burden of disease attributable to dietary PAH exposure, particularly in comparison to other major health risk factors such as smoking and alcohol consumption. In Singapore, smoking is linked to a 30% increased risk of cancer and a much more significant disease burden, contributing to a PAF of 0.036 and 13,000 DALY years for lung cancer^59^. While local statistics on alcohol and cancer risks were unavailable in this study, a recent report from the United States on the harmful effects of excessive alcohol consumption indicated that alcohol consumption is associated with a 13% increased risk of cancer, contributing to a PAF of 0.038 of all-cancer DALYs and 305,000 Years of Life Lost (YLL)^60^. Comparatively, cancer risk from dietary exposure to PAH is far smaller, between a 0.00463% and 0.517% increased risk of cancer. These findings suggest that even under the pessimistic scenario, dietary PAH exposure contributes only minimally to the overall cancer burden in Singapore. While methodological factors such as the behaviour of clustering algorithms used to classify exposure groups may introduce some uncertainty, the overall magnitude of the PAF remains substantially lower than that of major risk factors like smoking and alcohol consumption. Methodological rigour and analytical depth were ensured by Monte Carlo simulations with 100,000 iterations, which yielded stable exposure distributions and improved result reliability. By modelling both optimistic and pessimistic scenarios, the analysis effectively captured a broad range of potential exposure levels while accounting for uncertainties in the input data. The data covered information from 2,000 participants across 21 food categories stratified by age and ethnicity, providing a representative profile of the Singapore population. Additionally, examining the influence of multiple cooking methods on PAH levels added practical relevance to the findings. The risk assessment framework incorporated both lifetime cancer risk estimates and disease burden metrics through DALYs, allowing for meaningful comparisons with other public health risks.

However, several limitations should be considered when interpreting the results. The use of 24-hour recall data from the food consumption survey only reflected short-term dietary habits rather than longer-term patterns. While short-term exposure can provide useful insights, it may not fully account for individuals’ chronic or habitual consumption, which could be more strongly correlated with health outcomes^18^. Additionally, the study was unable to account for varying susceptibilities among different population subgroups or establish specific disease conditions associated with individual PAH consumption.

Secondly, the calculation of PAF in Eq. (5) may have underestimated the prevalence of dietary PAH exposure due to the inherent assumptions and limitations of the K-Means clustering method. K-Means is sensitive to outliers, as it minimizes the squared distance between data points and centroids. Outliers can disproportionately influence centroid placement during the iterative process, leading to suboptimal clustering. Additionally, the distribution of dietary PAH exposure was skewed towards lower values, meaning most individuals had relatively low exposure, while a few had significantly higher levels. Since K-Means assumes clusters with roughly equal variance and does not explicitly account for skewed distributions, it may face challenges in accurately separating exposed and unexposed groups. This could result in some lower-exposure individuals being classified as unexposed, or high-exposure outliers influencing centroid positions. Consequently, the proportion of the population classified as ‘exposed’ could be underestimated, potentially affecting the final PAF calculation.

Thirdly, only cancers were considered in the calculation of DALYs, which may not fully capture the range of potential sequelae associated with PAH exposure. Although a recent report has shown an increasing association between PAH exposure and cardiovascular disease (CVD), these risks are contextualised to environmental susceptibility through pollution and work-related exposure^61^. Existing literature regarding dietary exposure to PAHs offers limited insight into their broader health effects of PAHs beyond cancer^62^, and while the link between selected PAHs and carcinogenesis is well-documented in animal models, further evidence connecting PAH dietary exposure to human health outcomes remains lacking^16,63^. Furthermore, the analysis was limited to four PAH compounds (PAH4), which, while representative, may not capture the full spectrum of PAH exposure through diet.

Although the actual DALYs attributable to PAHs are likely to be higher than the values estimated in this study, it is not at an alarming level of concern as dietary exposure to PAHs carries a relatively lower risk compared to other routes of exposure. These compounds are typically much more concentrated in polluted air and urban environments, which pose a more direct and significant health risk^62^. This underscores the importance of prioritising risk mitigation strategies targeting exposure routes that can potentially lead to greater health impacts^47^. Additionally, the DALYs attributable to PAHs for the 2021 Singapore population, as estimated using the pessimistic model scenario for a conservative assessment, is at 92.5 years, which accounts for only 0.06% of the 158,418 DALYs associated with all cancers in 2021^64^. This represents a minimal contribution to the overall cancer burden, particularly when compared to major risk factors such as smoking, which alone contributes 18,343 DALYs for all cancers in Singapore^64^. These findings affirm that dietary PAH exposure is not a significant cause of concern relative to other preventable cancer risk factors in Singapore.

## Conclusion

Findings from this study showed that the current dietary exposure to PAHs in the Singapore population is low. While the carcinogenic potential of PAHs is established in animal models, evidence linking dietary PAH exposure to human health outcomes beyond cancer remains limited. Current dietary PAH exposure does not appear to be a significant public health concern. Although continued monitoring is prudent, risk mitigation strategies may be more effectively focused on other exposure routes for PAHs and other contaminants that are more prevalent in the food chain arising from climate changes^65^.

Further research is needed to assess PAH exposure in high-risk and vulnerable groups, particularly focusing on local population knowledge and consumer behaviours that may increase exposure risks. Understanding these factors would enable more targeted and effective public education campaigns for at-risk populations.

Extension of food safety exposure assessment to include human biomonitoring could be another approach for a more precise assessment of the distribution of risk in the population^66^. As PAH exposure stems from multiple sources, including smoking, alcohol consumption, occupational exposure, and environmental pollution, a comprehensive assessment approach using urinary biomarkers combined with dietary surveys could better quantify both total body PAH load and the specific contribution from food sources. This holistic understanding would help clarify the role of dietary exposure within overall PAH exposure patterns.

Research efforts should focus on developing and validating PAH reduction strategies in food processing and preparation, while ensuring thorough food safety assessments before implementation^67^. Policy considerations should include updating food safety guidelines, revising regulatory frameworks to account for cumulative PAH exposure, and developing targeted interventions for vulnerable populations. The establishment of a comprehensive monitoring system integrating food safety surveillance, consumer exposure assessment, and health outcome tracking would strengthen evidence-based policymaking. Additionally, economic analyses of intervention strategies and research into environmental factors affecting PAH formation, particularly in the context of climate change, would provide valuable insights for long-term food safety planning and resource allocation.

## Supplementary Information

Below is the link to the electronic supplementary material.

Supplementary Material 1

Supplementary Material 2

Supplementary Material 3

## Data Availability

All data generated or analysed during this study are included in this published article.
